# Supplementary material for: CTHRC1+ fibroblasts and SPP1+ macrophages synergistically contribute to pro-tumorigenic tumor microenvironment in pancreatic ductal adenocarcinoma
Source: Sci Rep. 2024 Jul 29;14:17412. doi: 10.1038/s41598-024-68109-z (PMC11286765; doi:10.1038/s41598-024-68109-z)
Supplement: Supplementary file 1 — Supplementary Figures. [file 41598_2024_68109_MOESM1_ESM.docx]

**Supplementary Figures**

**Fig. 1 Supplementary**

**a	b**

(A) Number of cells in each major cell population grouped by condition. (B) UMAP of B Cell populations.

**Fig. 2 Supplementary**

**a**

**b**

**c**

**d**

**e**

**f**

**g**

(A) Dot plot of stromal cell populations in the TME. (B) UMAP of matrix-associated genes enriched in *CTHRC1^+^GREM1*^+^ myCAF. (C) Heatmaps of top 25 DE genes enriched in *CTHRC1^+^GREM1*^+^ myCAF and proteoglycans. (D) TPM Expression of *CTHRC1* in the TCGA cohort, compared with all cancers. TPM of *CTHRC1* in tumor versus control in all cancers. (E) Bar plot of upregulated KEGG enrichment pathways in *CTHRC1^+^GREM1*^+^ myCAF using Enrichr. (F) UMAP of matrix, matrix remodeling, and angiogenesis enrichment signatures. Violin plot of EMT enrichment signature. (G) Additional GSEA plots of upregulated pathways in *CTHRC1^+^GREM1*^+^ myCAF.

**Fig. 3 Supplementary**

**a**

**b**

**c**

**d**	*SPP1^+^APOE*^+^ TAM	*SPP1^+^VEGFA^+^* TAM

**e**

(A) Dot plot of myeloid cell populations in the TME. (B) UMAP of enriched genes expressed by *SPP1*^+^ macrophages. (C) UMAP of matrix, matrix remodeling, hypoxia, M1 and M2 signatures, and pro-tumor cytokine enrichment signatures. (D) Bar plot of upregulated KEGG pathways in *SPP1^+^APOE*^+^ TAM. Bar plots of upregulated MSigDB and KEGG pathways in *SPP1^+^VEGFA*^+^ TAM. (E) GSEA plots of enriched pathways in *SPP1^+^APOE*^+^ TAM.

**Fig 4 Supplementary**

**a**	*GREM1*	*APOE*

(A) Expression box plots of *GREM1* and *APOE* gene expression in control versus tumor patients (TCGA cohort).

**Fig. 5 Supplementary**

**a**

**b**

**c**

**d**

(A) Dot plot of spatial cell populations in the TME (Patient 1). (B) Spatial expression plots of matrix-associated genes and immune cell canonical markers. (C) Spatial enrichment of *SPP1^+^APOE*^+^ TAM, *CTHRC1^+^GREM1*^+^ myCAF, and EMT signatures in patients 2 and 3. (D) Top Liana ligand-receptor interactions of *CTHRC1^+^GREM1*^+^ myCAF and *SPP1*^+^ TAM.

**Fig. 6 Supplementary**

**a**

(A) Bar plot of top GSEA terms from *ITGB1* KO perturbed genes. Egocentric plot of *ITGB1* KO perturbed genes, along with corresponding GSEA terms.
